# PIMKL: Pathway-Induced Multiple Kernel Learning

**DOI:** 10.1038/s41540-019-0086-3

**Published:** 2019-03-05

**Authors:** Matteo Manica, Joris Cadow, Roland Mathis, María Rodríguez Martínez

**Affiliations:** 1 IBM Research, Zürich, Switzerland; 2 ETH, Zürich, Switzerland

## Abstract

Reliable identification of molecular biomarkers is essential for accurate patient stratification. While state-of-the-art machine learning approaches for sample classification continue to push boundaries in terms of performance, most of these methods are not able to integrate different data types and lack generalization power, limiting their application in a clinical setting. Furthermore, many methods behave as black boxes, and we have very little understanding about the mechanisms that lead to the prediction. While opaqueness concerning machine behavior might not be a problem in deterministic domains, in health care, providing explanations about the molecular factors and phenotypes that are driving the classification is crucial to build trust in the performance of the predictive system. We propose Pathway-Induced Multiple Kernel Learning (PIMKL), a methodology to reliably classify samples that can also help gain insights into the molecular mechanisms that underlie the classification. PIMKL exploits prior knowledge in the form of a molecular interaction network and annotated gene sets, by optimizing a mixture of pathway-induced kernels using a Multiple Kernel Learning (MKL) algorithm, an approach that has demonstrated excellent performance in different machine learning applications. After optimizing the combination of kernels to predict a specific phenotype, the model provides a stable molecular signature that can be interpreted in the light of the ingested prior knowledge and that can be used in transfer learning tasks.

## Interpretable classification of biomedical samples with PIMKL

The reliable classification of biomedical samples to predict phenotypic differences requires not only robust methods, but also interpretable approaches that can explain the reasons behind a prediction. A team led by María Rodríguez Martínez at IBM Research - Zürich has developed PIMKL, a methodology that exploits prior knowledge and enables the integration of multiple types of data with varying predictive power. Even when noisy datasets are simultaneously analyzed, PIMKL is able to discard uninformative data and achieve strong prediction power. Importantly, PIMKL produces a molecular signature that enables the interpretation of the results in terms of known biological functions. This signature can be transferred to other cohorts without loss of performance, demonstrating surprising robustness across cohorts. Interpretable algorithms can effectively help gain insights about disease mechanisms and build trust in a model.

## Introduction

Designing reliable and interpretable predictive models for patient stratification and biomarker discovery is a daunting challenge in computational biology. A plethora of methods based on molecular data have been proposed throughout the years, many of which exploit prior knowledge about the molecular processes involved in the regulation of the phenotype to be predicted. Prior knowledge is frequently encoded as a molecular interaction network, where nodes represent genes or proteins and edges represent relationships between the connected nodes. Supporting the development of such methods, the number of databases reporting protein-protein interactions has seen an unprecedented growth in recent years, and databases such as STRING,^1^ OmniPath,^2^ Reactome,^3,4^ IntAct,^5^ MINT,^6^ MatrixDB,^7^ HPRD,^8^ KEGG,^9–11^ or Pathway Commons,^12^ just to name a few, provide an incredibly useful resource to design models informed about the underlying molecular processes.

Several studies have focused on comparing prior knowledge-based classification methods. For instance, Cun and Fröhlich^13^ evaluated 14 machine learning approaches to predict the survival outcome of breast cancer patients. The methods included among others: average pathway expression,^14^ classification by significant hub genes,^15^ pathway activity classification,^16^ and a series of approaches based on Support Vector Machines (SVMs), such as network-based SVMs,^17^ recursive feature elimination SVMs,^18^ and graph diffusion kernels for SVMs.^19,20^ The study concluded that, while none of the evaluated approaches significantly improved classification accuracy, the interpretability of the gene signatures obtained was greatly enhanced by the integration of prior knowledge.

A more recent benchmarking effort was provided by a collaboration between the National Cancer Institute (NCI) and the Dialogue on Reverse Engineering Assessment and Methods (DREAM) project.^21^ The NCI-DREAM challenge aimed to identify the top-performing methods to predict therapeutic responses in breast cancer cell lines using genomic, proteomic, and epigenomic data profiles. A total of 44 prediction algorithms were scored against an unpublished and hidden gold-standard dataset. Two interesting conclusions emerged from the challenge. First, all top-performing methods modeled nonlinear relationships and incorporated biological pathway information, and second, performance was increased by including multiple, independent datasets. Interestingly, the top-performing methodology, Bayesian Multitask Multiple Kernel Learning, exploited a multiple kernel learning (MKL) framework.^22^

MKL methods aim to model complex and heterogeneous datasets by using a weighted combination of base kernels. While in more traditional kernel methods the parameters of a single kernel are optimized during training, in MKL, the weights of all kernels are tuned together during training. Compared to single-kernel methods, the advantages of MKL are two-fold. First, different kernels can encode various levels of information, e.g., different definitions of similarity or different types of data, endowing the algorithm with the flexibility required to model heterogeneous or multi-modal datasets. Second, after optimizing the combination of kernels, the weights associated with each kernel can provide valuable insights about the sets of features that are most informative for the classification task at hand.

In this paper, we seek to augment the predictive power and interpretability of MKL methods, by enhancing them with the use of prior knowledge. Towards this end, we introduce the Pathway-Induced Multiple Kernel Learning (PIMKL), a supervised classification algorithm for phenotype prediction from molecular data that jointly exploits the benefits of MKL and prior knowledge ingestion. PIMKL uses an interaction network and a set of annotated gene sets to build a mixture of pathway-induced kernels from molecular data, whose mixture is then optimized with an MKL algorithm. After PIMKL is trained, the weight assigned to each kernel provides information about the importance of the corresponding pathway in the mixture. As a result, a molecular signature characterizing the phenotype of interest is derived.

While there are currently many approaches that take advantage of the known graph structure of a molecular system,^19,23^ or use collections of annotated gene sets as prior knowledge to reduce the dimensionality of molecular profiles and enable the analysis of tumor profiles,^24,25^ to our knowledge PIMKL is the first methodology that integrates both levels of prior knowledge—molecular networks and collections of pathways—with state-of-the-art machine learning approaches. We demonstrate that the use of MKL enhances the classification performance, and the use of prior knowledge ensures that the results are interpretable, while shedding light on the molecular interactions implicated in the phenotype.

This paper is structured as follows. We first describe PIMKL and validate it by predicting disease-free survival for breast cancer samples from multiple cohorts. We benchmark PIMKL by comparing it with the methods analyzed in.^13^ To evaluate its generalization power, we use a PIMKL-generated molecular signature to predict disease-free survival on a different dataset, the METABRIC breast cancer cohort.^26^ Finally, we examine PIMKL robustness against noise and test its capabilities to integrate distinct data types by simultaneously using METABRIC gene expression (mRNA) and copy number alteration (CNA) data for the same classification task. Our analysis suggests that PIMKL provides an extremely robust approach for the integration of multiple types of data with prior knowledge that can be successfully applied to a wide range of phenotype prediction problems.

## Results

In the following sections, we discuss the application of PIMKL to different breast cancer cohorts. First, in Section 2.1, PIMKL is compared to a previous study by Cun and Frölich^13^ where different algorithms for phenotype prediction and gene selection using prior knowledge were compared. Later, in Section 2.2, PIMKL is applied to gene expression and copy number data from the METABRIC cohort^26^ with two purposes: first, we aim to test whether transfer learning between different studies is possible, and, second, we want to evaluate PIMKL performance in the analysis of multi-omics analysis in the presence of noise or uninformative data. Regarding evaluation plots, all box plots are constructed in a similar manner: the box reports the first and the third quartile; the median is reported as a horizontal line inside the box; and the whiskers represent the most extremal data within 1.5 times the IQR (interquartile range) below and above the box.

### PIMKL on breast cancer microarray cohorts

PIMKL was tested on microarray gene expression data from six breast cancer cohorts (see Supplementary Table S1 for details about the cohorts). The classification task consisted in stratifying breast cancer samples according to occurrence of relapse within 5 years. To ensure the fairest possible comparison, we used the same interaction sources as in the study by Cun and Fröhlich, namely a merge between KEGG pathways and Pathway Commons. As access to the older release of KEGG is restricted, the most recent versions from both sources were used. A collection of 50 *hallmark* gene sets from the Molecular Signatures Database (MSigDB) version 5.2^27^ was used to define the sub-graphs used for pathway induction, generating *P* = 50 kernels. The classification performance was evaluated by means of the Area Under the receiver operating characteristic Curve (AUC). We closely followed the same data processing procedures and the cross-validation scheme as proposed in the original study (for details, see Supplementary Algorithm S1).

**Fig. 1:**
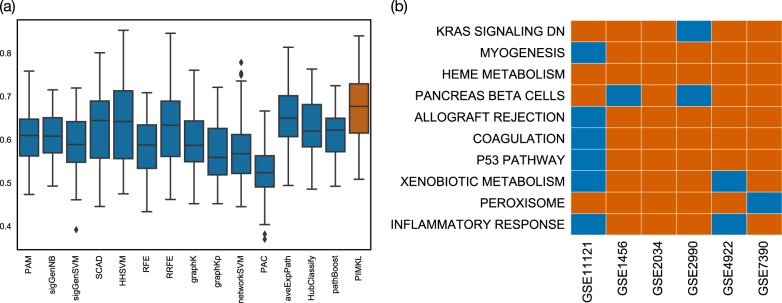
PIMKL cross-validation results. **a** Box plots for AUC values over all cohorts for the methods considered. PIMKL results are reported in red, while other methods results are colored in blue. Box plots are obtained from ten (repeats of) mean AUC values over 10-fold cross-validation splits, see Algorithm S1. **b** Heat map showing significant pathways selected by PIMKL across the different cohorts considered in the study. Significant pathways are highlighted in red, while non-significant are colored in blue

The results of PIMKL compared to the 14 algorithms considered by Cun and Fröhlich are reported in Fig. 1. Overall AUC values for the 6 cohorts over the cross-validation rounds for all considered methods are shown in Fig. 1a. AUC values for the single cohorts can be found in Supplementary Fig. S1, where PIMKL exhibits the highest median value and consistently outperforms the other methods or is in the top performers group on single cohorts.

As discussed in Section 4, PIMKL generates a molecular signature given by the weighted contribution of each kernel. Each weight represents the relative importance of each hallmark pathway used for pathway induction to explain the phenotype. To evaluate the stability of the signature, the pathway weight distribution over cross-validation rounds was analyzed. Our baseline stands for the case where all kernels have the same weight: $w_{b} = \frac{1}{P}$, representing a situation where no pathway contributes more than the others to the phenotype prediction. To find whether a pathway is significant for the phenotype, the distribution of the kernel weights with median above *w*_*b*_ was tested against the baseline using a one-sample Wilcoxon signed-rank test. *p*-values at significance level 0.001 were corrected for multiple testing using Benjamini–Hochberg. Pathways where significance was achieved in at least four of six cohorts are reported in Fig. 1b. Furthermore, Supplementary Fig. S2 shows the box plots of the weights over cross-validation runs of the top-30 significant pathways on all the 6 cohorts. We note that the gene sets that consistently had the highest weights in all cohorts are well-established cancer pathways: KRAS signaling, P53 pathway, MYC targets, etc, suggesting once again that the selection of these signatures was not due to random chance. Supplementary Fig. S3 provides a summarized view of the significant pathways across cohorts.

Interestingly, heme metabolism pathway was significant in all cohorts. This pathway is involved in the metabolism of heme and erythroblast differentiation. A possible explanation is that heme metabolism might reflect an active vascularization of the samples, a phenomenon widely observed in cancer progression.^28^ A more intriguing hypothesis is a possible association between elevated heme metabolism and cancer progression, as has been reported in non-small-cell lung cancer cells and xenograft tumors.^29^ It is also interesting to look at the pathways that are significant in at least five cohorts: KRAS signaling, myogenesis, allograft rejection, coagulation, P53 pathway, and peroxisome. All of these pathways are associated with breast cancer. For instance, activation of KRAS signaling has been reported to promote the mesenchymal features of basal-type breast cancer.^30,31^ Myogenesis, or the process of formation of muscular tissue, is commonly disrupted in cancer.^32^ Allograft rejection might reflect an immune-mediated tumor rejection signature following administration of immunotherapeutic agents.^33^ Several studies have suggested a role for blood coagulation proteins in tumor progression.^34–36^ P53 is the most commonly mutated protein in cancer.^37,38^ Finally, peroxisomes are small, membrane-enclosed organelles that contain enzymes involved in a variety of metabolic reactions, including several aspects of energy metabolism. Altered peroxisome metabolism has been linked to various diseases, including cancer.^39,40^

**Fig. 2:**
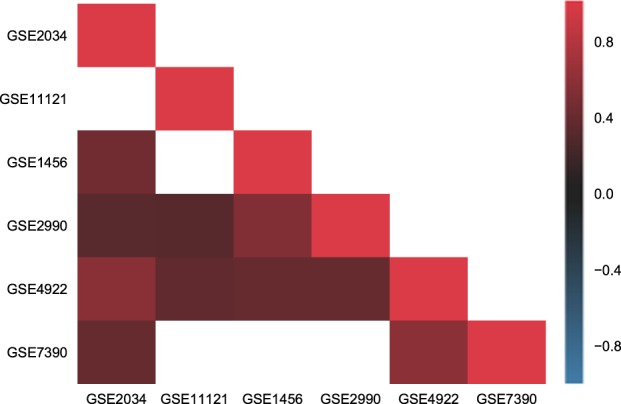
Correlation in molecular signatures. Heat map reporting the correlation of the molecular signature estimated across multiple cohorts. Correlation values are reported in the lower triangular part of the heat map (since it is symmetric) on blue to red scale, where white squares indicate non-significant correlations. All cohorts exhibit a positive correlation, significant in most cases, proving the stability of the molecular signature obtained with PIMKL

Figure 2 reports the correlation of the PIMKL molecular signatures estimated across multiple cohorts and highlights their stability across different studies, suggesting that a cohort-independent disease-free survival signature for breast cancer has been learned.

**Fig. 3:**
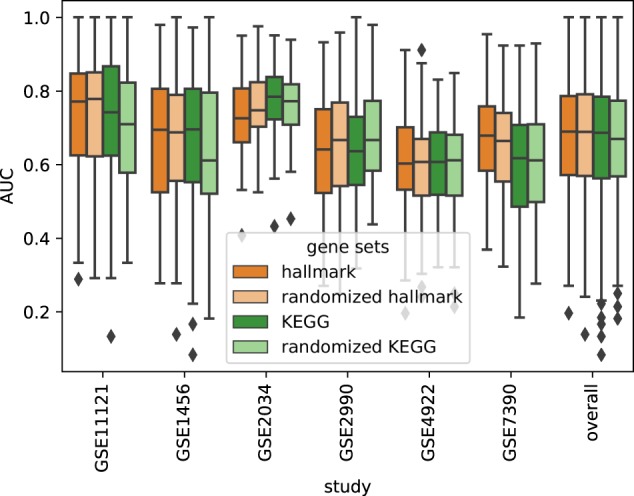
PIMKL cross-validation AUC for different gene sets. Box plots of all 100 AUC values (overall 600) for pathway-induced MKL obtained by Algorithm S1 with different gene sets to define the pathways given the same aforementioned interactions. In addition to the 50 previously introduced hallmark gene sets, results for 186 KEGG gene sets from the Molecular Signatures Database (MSigDB) version 5.2^27^ and also respective randomized gene sets are reported. For randomization, the same number of gene sets was created, each set with random size between 50 and 250 genes by sampling from the union of all gene sets. The quartiles are comparable within each cohort proving the stability of the methods towards gene sets selection

Importantly, results were consistent when other gene sets were used. For instance, PIMKL exhibited performance robustness, in terms of AUC, when we considered gene sets of different size and even when we used randomized versions of functionally related gene sets (see Fig. 3). We note that robustness against pathway size variability is expected, as we employ the trace normalization to generate the kernels (see Section 4.1), which makes kernels generated from different gene sets comparable and mitigates size effects.

Robustness when considering randomized versions of functional gene sets demonstrates that PIMKL performance does not depend on the specific selection of pathways, and that through the MKL optimization we can identify informative gene sets in disparate collections of genes. Notice, however, that while choosing random gene sets does not worsen PIMKL performance, interpretation of the molecular signatures, as we will discuss next, is only possible when the sets have a well-defined biological function.

### PIMKL on METABRIC cohort

To test PIMKL applicability to multi-modal datasets, we used our methodology to predict disease-free survival in the METABRIC breast cancer cohort, consisting of 1890 samples profiled with Illumina Human v3 microarray data (mRNA) and Affymetrix SNP 6.0 copy number data (CNA), see Supplementary Table S2 for details.

**Fig. 4:**
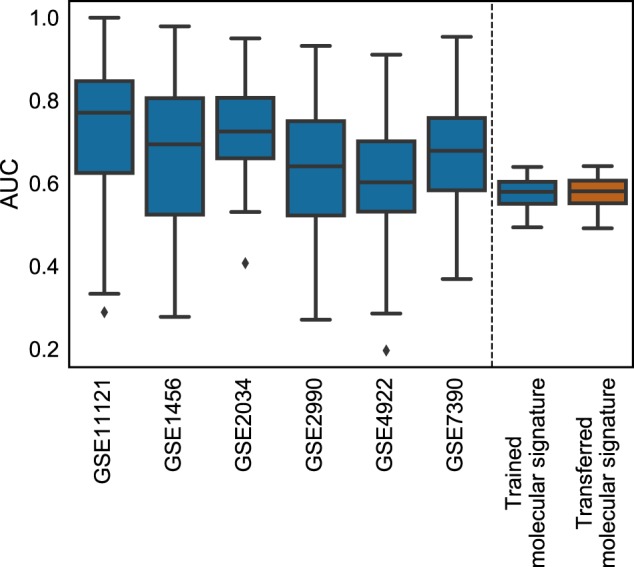
PIMKL performance on METABRIC. Box plots of the performance of PIMKL over the six cohorts used to benchmark the method (left of the dashed vertical line) and its application on METABRIC for disease-free survival prediction (right of the dashed vertical line). Optimized weights at training by EasyMKL (blue); provided weights from taking the pathway-wise median weights of the six signatures obtained during benchmarking (red)

In order to validate the generalization power of PIMKL-generated molecular signatures, we first focused on the analysis of METABRIC microarray data. Our hypothesis here is that the underlying molecular mechanisms associated with disease-free survival are the same in different cohorts and, as such, knowledge learned in one cohort can be transferred to another one. After computing the pathway-induced kernels with the same procedure adopted in Section 2.1, a set of pathway weights was defined using the median of the weights obtained in the six previously analyzed cohorts. Figure 4 shows the results obtained by training a KOMD classifier using the weights transferred from the six independent cohorts and by learning METABRIC-specific pathway weights (for details see Supplementary Algorithm S2). It is evident that both molecular signatures perform very similarly. Indeed, the two signatures are highly correlated (Pearson correlation $\rho = 0.72$, *p*-value = $3.34 \cdot 10^{ - 9}$, Supplementary Fig. S4). It is important to notice that the variance of the prediction results is also consistently reduced, probably due to the newer microarray technology used by the METABRIC study.

To test PIMKL’s capability to integrate multi-omics data, both the mRNA and CNA data from the METABRIC cohort were jointly utilized in the same predictive task. A set of additional kernels was generated using the copy number data and then used in two ways: first, the CNA kernels were independently optimized with PIMKL, and second, a mixture of CNA and mRNA kernels were jointly optimized.

**Fig. 5:**
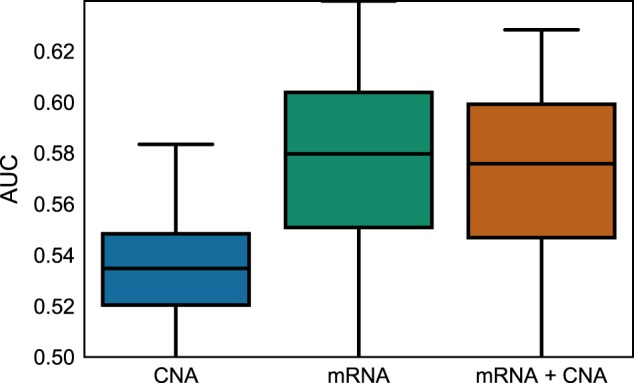
PIMKL performance on METABRIC multi-omics. Box plots for AUC values obtained applying PIMKL on different data types and their integration. Results based on CNA data alone are reported in blue, results based on RNA data alone are reported in green and results based on the integration of CNA and RNA data are repored in red

From Fig. 5, it is evident that the CNA data were not as predictive as mRNA regarding disease-free survival. However, it is interesting to notice that PIMKL was able to discard noisy kernels—associated with CNA data—to achieve similar levels of performance when using the more informative mRNA data alone and when using a mixture of CNA and mRNA data. This suggests that the application of the proposed algorithm is feasible even when no prior knowledge about the information content of each single omic type is available.

## Discussion

We have presented here PIMKL (Pathway-Induced Multiple Kernel Learning), a novel, effective and interpretable machine learning methodology for phenotype prediction using multi-modal molecular data. PIMKL is based on a multiple kernel learning (MKL) framework, a kernel-based method that has demonstrated excellent capabilities to integrate multi-omics datasets.^21^ In addition, PIMKL also exploits prior knowledge in the form of molecular interaction networks and sets of annotated pathways with known biological functions to build a mixture of pathway-induced kernels. The main novelty introduced in this work is the definition of multiple interaction-aware kernel functions, which enables us to encode information about the molecular prior knowledge related to a phenotype, and facilitates the interpretation of the results in terms of known biological functions and/or specific molecular interactions. We achieved this by using kernels to map samples into the space of network edges, i.e., molecular interactions, recovering a direct biological interpretation. The kernel weights are later optimized to classify a phenotype or a clinical variable of interest.

In this work, PIMKL was extensively tested in the context of predicting disease-free survival from breast cancer samples. We have demonstrated that the resulting weighted combination of kernels can be interpreted as a phenotypic molecular signature and provides insights into the underlying molecular mechanisms. As a benchmark, a well-studied set of cohorts, previously analyzed using a range of stratification methods, has been adopted.^13^ The quality and the stability of the obtained signatures has been thoroughly investigated, and we have shown that PIMKL outperforms other methods and finds stable molecular signatures across different breast cancer cohorts. Despite outperforming other methods, it could be argued that the achieved performance is relatively modest. We would like to point out that predicting survival, or the more commonly used recurrent free survival, using a unique source of noisy, high-throughput data measured at a single time point is an incredibly difficult, if not impossible, task. Since many important layers of regulation that affect gene and protein expression are not captured in the transcriptomic profiles, a high AUC curve should not be expected. In addition, the data used by Cun and colleagues^13^ and reused by us consist of 6 studies published between 2005–2008 that used a relatively old microarray technology (see Table S1 for details). More modern high-throughput technologies are expected to result in better AUC values. For instance, preliminary analyses on multi-omics cohorts consisting of RNAseq, CNA and high-throughput proteomic data in prostate cancer have demonstrated median AUC values over 0.95. Similarly, analyses of proteomic datasets to predict tumor recurrence status after 5 years using PIMKL have resulted in median AUC over 0.85, compatible with the usage of the algorithm in a clinical setting.

In this work, we also investigated the generalization power of the found signatures by testing them on unseen mRNA breast cancer data from the METABRIC cohort and the associated disease-free survival data. The obtained results confirmed that the algorithm can be used to effectively gain insights into disease progression and that this knowledge can be transferred to other cohorts without loss of performance. Furthermore, PIMKL can be seamlessly applied to integrate data from different omic layers. Its intrinsic capability to discard noisy molecular features has been demonstrated by applying it on METABRIC, where it was possible to integrate multiple types of data with varying predictive power. Even when non-informative data was mixed with informative data, PIMKL was able to discard uninformative kernels and achieve similar levels of performance. Evidently, PIMKL is not restricted to breast cancer, to the specific omic data types or to the sources of prior information used in this work. Its application is open to other disease types using any available combination of data together with any suitable prior network and sets of genes.

Besides being capable of using different types of prior knowledge, the proposed approach is also highly flexible with regard to the number and nature of the selected kernels. Indeed, PIMKL was developed by making use of an efficient implementation of EasyMKL,^41^ an extremely scalable MKL algorithm with constant memory complexity independent of the number of kernels. This efficiency can potentially allow the user to define smaller pathways, leading to a more fine-grained characterization and understanding of the molecular mechanisms involved in disease progression with limited performance drawbacks.

Finally, possible extensions of PIMKL, such as optimizing the kernel mixture using semi-supervised or unsupervised multiple kernel learning methodologies,^42^ may help to discover phenotype-independent pathway signatures and will be explored in the future. To summarize, PIMKL provides a flexible and scalable method to translate prior knowledge and molecular data into actionable insights in a clinical setting.

## Methods

PIMKL is a methodology for phenotype prediction from multi-omic measurements, e.g., mRNA, CNA, etc, based on the optimization of a mixture of pathway-induced kernels. Such kernels are generated by exploiting prior knowledge in a dual fashion. First, prior knowledge is injected in PIMKL in the form of a molecular interaction network, and second, as a set of annotated gene sets or pathways.

**Fig. 6:**
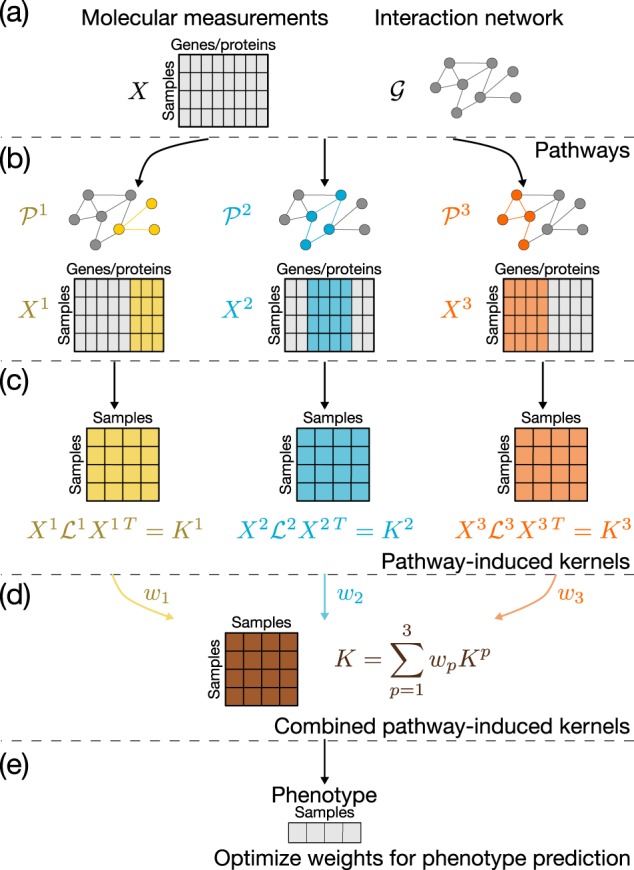
PIMKL concept. **a** Given measurements for a set of molecular entities and a network topology describing their interactions, **b** relevant sub-networks and data subsets can be extracted using pathway annotations, **c** to generate a mixture of pathway-induced kernels. **d** These kernels can be combined using a set of weights, **e** that are optimized to predict a phenotype of interest. The weights of the mixture provide a measurement of the importance of each pathway, thereby shedding light on the molecular mechanisms that contribute to the phenotype

A key aspect of PIMKL is pathway induction, a method to generate similarity functions using the topological properties of an interaction network. In practice, we use pathway gene sets with well-defined biological functions to define sub-networks from which we generate pathway-induced kernels. The mixture of pathway-induced kernels is then optimized to classify a phenotype of interest, and in doing so, each pathway is assigned a weight representing its importance to explain the phenotype. The established link between kernels and pathways enables PIMKL to identify which molecular mechanisms are important for the prediction of the considered phenotype. Figure 6 summarizes PIMKL’s approach. First, we consider measurements of a collection of molecular entities, where the interactions between the entities are extracted from a prior knowledge molecular topology (Fig. 6a). Next, we use pathway annotations to generate a relevant set of sub-networks and associated measurements (Fig. 6b). We transform the collection of sub-networks into a collection of kernels using a pathway induction procedure (see details in Section 4.1), and combine the resulting kernels as a weighted mixture of kernels (Fig. 6c, d). Finally, the mixture is optimized to predict a specific phenotype and the weight associated with each kernel is interpreted as the importance of that pathway towards the prediction of the phenotype (Fig. 6e).

### Pathway Induction

PIMKL encodes information from the topology of each pathway’s sub-network. The approach of integrating pathway information into interaction-aware kernel similarity functions is here termed pathway induction. Specifically, we design kernel functions by utilizing a positive semidefinite (PSD) matrix that encodes the topological properties of a graph. Given any PSD matrix *M*, a valid kernel can be induced through the following weighted inner product^43^:

1 $$k(x,y) = x^TMy.$$

This ensures the existence of a matrix *U*:

2 $$M = U^TU,$$

3 $$\phi (x) = Ux,$$

where *ϕ* is a mapping describing a transformation in the feature space. By making use of a PSD matrix encoding the topological properties of a graph representing a pathway, it is possible to design interaction-aware kernels. For instance, let us consider an undirected graph representing a pathway:

4 $${\cal P} = (V,E),$$

with $N_v = |V|$ nodes and $N_e = |E|$ edges representing the genes/proteins and their interactions respectively. Such a graph is defined by a symmetric adjacency matrix $A \in \{ 0,1\} ^{N_v \times N_v}$:

5 $$A_{ij} = 1\,\forall (i,j) \in E,$$

and a diagonal degree matrix $D \in {\Bbb R}^{N_v \times N_v}$:

6 $$D_{jj} = \mathop {\sum}\limits_i {\kern 1pt} A_{ij}.$$

For such a graph, we can compute a Laplacian matrix $L \in {\Bbb R}^{N_v \times N_v}$ as follows:

7 $$L = D - A.$$

The Laplacian is a PSD matrix and therefore represents a suitable candidate for induction of a weighted inner product based on a pathway topology. This can be shown by defining an ordered incidence matrix $S \in {\Bbb R}^{N_v \times N_e}$ for ${\cal P}$ that, by construction, satisfies the relation $L = SS^T$. After introducing an index set ${\cal E}$ for the edges *E*, *S* can be defined as:^44^

8 $$S_{ne} = \left\{ {\begin{array}{*{20}{l}} 1 \hfill & {{\mathrm{if}}\,n = i \wedge i \le j} \hfill \\ { - 1} \hfill & {{\mathrm{if}}\,n = j} \hfill \\ 0 \hfill & {{\mathrm{otherwise}},} \hfill \end{array}} \right.$$

9 $${\mathrm{where}}\,e \in {\cal E}\,{\mathrm{corresponds}}\,{\mathrm{to}}\,{\mathrm{edge}}\,(i,j) \in E\,{\mathrm{and}}\,n \in V.$$

Moreover, the Laplacian can be interpreted as a discrete Laplace operator. Indicating with $X \in {\Bbb R}^{N \times N_v}$ a set of *N* samples, a discrete diffusion process over graph nodes can be described as:

10 $$LX^T = SS^TX^T,$$

where the term *S*^*T*^*X*^*T*^ computes the discrete diffusion potential along the edges, and Eq. 10 describes how the flow of this potential is updated when a node’s incoming and outgoing flows are aggregated.

Decomposing the Laplacian using an ordered incidence matrix is equivalent to mapping the samples *X* from the original space with measurements of *N*_*v*_ molecular entities into an *N*_*e*_-dimensional feature space, where each pathway interaction is a dimension and the value along the edge is the discrete diffusion potential between the respective node’s measurements. The inner product in this space is a similarity function, or kernel *k*_*L*_(*x*, *y*), defined as:

11 $$k_L(x,y) = x^TLy = x^TSS^Ty\,\forall x,y \in {\Bbb R}^{N_v}.$$

Similar considerations can be applied to weighted graphs with non-negative weights. Given a weighted undirected graph ${\cal P} = (V,E,W)$ and $W \in {\Bbb R}^{N_e \times N_e}$ an associated diagonal weights matrix, the Laplacian *L* is defined as:

12 $$L = SWS^T$$

13 $$L_{ij} = \left\{ {\begin{array}{*{20}{l}} {d_i - W_e} \hfill & {{\mathrm{if}}\,i = j} \hfill \\ { - W_e} \hfill & {{\mathrm{otherwise}},} \hfill \end{array}} \right.$$

14 $${\mathrm{where}}\,e \in {\cal E}\,{\mathrm{corresponds}}\,{\mathrm{to}}\,{\mathrm{edge}}\,(i,j) \in E\,{\mathrm{and}}\,d_{i}\,{\mathrm{is}}\,{\mathrm{the}}\,{\mathrm{degree}}\,{\mathrm{of}}\,{\mathrm{node}}\,i{.}$$

To ensure an equal contribution from all the nodes in the considered pathway, the degree-normalized version of the Laplacian ${\cal L}$ can be adopted:

15 $${\cal L} = D^{ - \frac{1}{2}}SWS^TD^{ - \frac{1}{2}}$$

16 $${\cal L}_{ij} = \left\{ {\begin{array}{*{20}{l}} {1 - \frac{{W_e}}{{d_i}}} \hfill & {{\mathrm{if}}\,i = j\,{\mathrm{and}}\,d_i \ne 0} \hfill \\ { - \frac{{W_e}}{{\sqrt {d_id_j} }}} \hfill & {{\mathrm{if}}\,i\,{\mathrm{and}}\,j\,{\mathrm{are}}\,{\mathrm{adjacent}}} \hfill \\ 0 \hfill & {{\mathrm{otherwise}},} \hfill \end{array}} \right.$$

17 $${\mathrm{where}}\,e \in {\cal E}\,{\mathrm{corresponds}}\,{\mathrm{to}}\,{\mathrm{edge}}\,(i,j) \in E\,{\mathrm{and}}\,d_{i}\,{\mathrm{is}}\,{\mathrm{the}}\,{\mathrm{degree}}\,{\mathrm{of}}\,{\mathrm{node}}\,i{.}$$

This pathway encoding directly leads to the definition of pathway induction used in this work. Given any two samples measurement *x*, $y \in {\Bbb R}^{N_v}$:

18 $$\begin{array}{*{20}{l}} {k_{\cal L}(x,y)} \hfill & = \hfill & {x^T{\cal L}y = } \hfill \end{array}$$

19 $$\begin{array}{*{20}{l}} {} \hfill & = \hfill & {x^TD^{ - \frac{1}{2}}SWS^TD^{ - \frac{1}{2}}y = x^T\left( {D^{ - \frac{1}{2}}SW^{\frac{1}{2}}} \right){\kern 1pt} \left( {W^{\frac{1}{2}}S^TD^{ - \frac{1}{2}}} \right)y = } \hfill \end{array}$$

20 $$\begin{array}{*{20}{l}} {} \hfill & = \hfill & {x^T{\cal S}{\cal S}^Ty = {\mathrm{{\Pi}}}(x)^T{\mathrm{{\Pi}}}(y),} \hfill \end{array}$$

with:

21 $${\mathrm{{\Pi}}}(x) = \left\{ {\begin{array}{*{20}{l}} {\sqrt {W_e} \frac{{x_i}}{{\sqrt {d_i} }}} \hfill & {{\mathrm{if}}\,i = j\,{\mathrm{and}}\,d_i \ne 0} \hfill \\ {\sqrt {W_e} \left( {\frac{{x_i}}{{\sqrt {d_i} }} - \frac{{x_j}}{{\sqrt {d_j} }}} \right)} \hfill & {{\mathrm{if}}\,i\,{\mathrm{and}}\,j\,{\mathrm{are}}\,{\mathrm{adjacent}}} \hfill \\ 0 \hfill & {{\mathrm{otherwise,}}} \hfill \end{array}} \right.$$

22 $${\mathrm{where}}\,e \in {\cal E}\,{\mathrm{corresponds}}\,{\mathrm{to}}\,{\mathrm{edge}}\,(i,j) \in E\,{\mathrm{and}}\,d_{i}\,{\mathrm{is}}\,{\mathrm{the}}\,{\mathrm{degree}}\,{\mathrm{of}}\,{\mathrm{node}}\,i{.}$$

A similar concept was proposed in^45^ using a full network set, instead of pathway-associated sub-networks. The normalized Laplacian was used as a regularizer to constrain the optimization problem when training an SVM. In PIMKL, we arrive at a similar formulation of the problem by introducing a feature mapping instead of using the Laplacian as a regularizer. We define a kernel function that allows easy application to any kernelized method and any further kernel transformation, e.g., polynomial, Gaussian, etc. The decomposition of ${\cal L}$ can be derived from the graph but is implicit, and can be easily extended to the multiple kernel learning case, allowing us to work at a pathway/sub-network level.

It should be noted that in PIMKL, the individual pathway-induced kernels are set to have equal trace (equal average self similarity of the samples) to learn fair relative weights independent of the sub-network/gene set size.

**Fig. 7:**
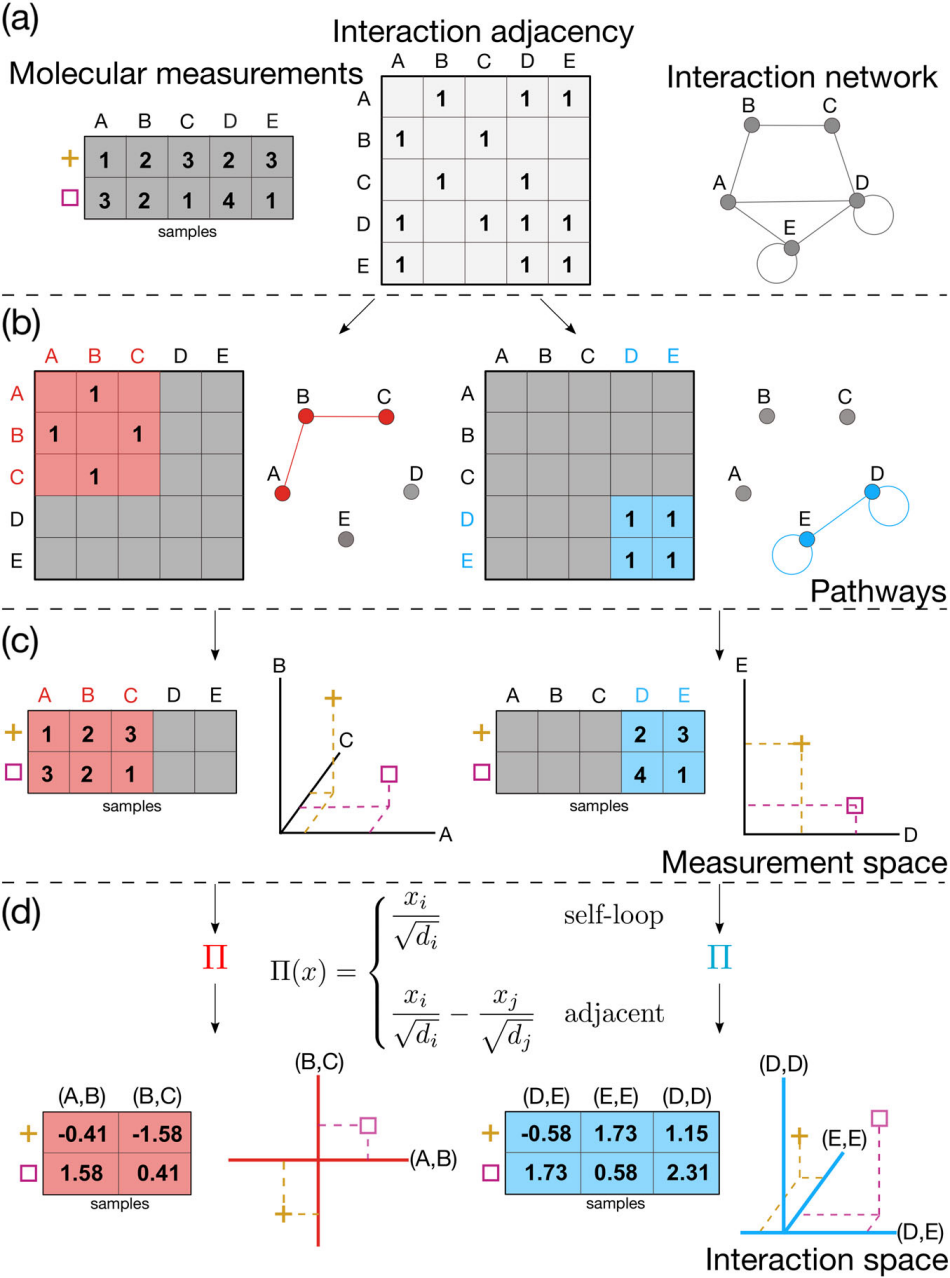
Pathway induction. **a** PIMKL exploits sets of interacting entities with molecular measurements on different samples for which we know the network connectivity and adjacency matrix; **b** using pathway annotations, we can map individual sample measurements from their original space, where each entity is a node (**c**), to the space of the interactions between molecular entities (**d**). The example above shows how the mapping using pathway induction transforms the considered samples using two different pathways

A schematic illustration of the mapping introduced using pathway induction can be observed in Fig. 7. The molecular measurements and the complete interaction network with its adjacency matrix depicted in Fig. 7a can be combined with the information from the gene sets to extract sub-networks (Fig. 7b) and the related selection of measurements (Fig. 7c). Using pathway induction, as described above, we can map the samples from the measurement space to the interaction space, thereby obtaining a data representation where interactions between the molecular entities are taken into consideration.

### Pathway-Induced Multiple Kernel Learning

PIMKL makes use of the concept of pathway induction, defined in 4.1, to implement a multiple kernel learning classification system. Consider a network that recapitulates a comprehensive set of known molecular interactions represented by a graph ${\cal G} = (V,E,W)$ with $N_v = |V|$ nodes, $N_e = |E|$ edges and a set of molecular measurements $X \in {\Bbb R}^{N \times N_v}$ with associated labels for a relevant phenotype *y*.

Given a selection of pathways *P*, e.g., gene sets from ontologies or inferred via community detection, it is possible to extract for each pathway $p \in P$, a corresponding sub-graph ${\cal P}^p = (V^p,E^p,W^p) \subset {\cal G}$ with $N_v^p = |V^p|$ nodes, $N_e^p = |E^p|$ edges and a sub-selection of measurements corresponding to the genes contained in the pathway $X^p \in {\Bbb R}^{N \times N_v^p}$.

For every pathway, a Gram matrix *K*^*p*^ can be used to represent the pathway-induced kernel, where *K*^*p*^ is computed for each pair of samples *i* and *j* as follows:

23 $$K_{ij}^p = k_{{\cal L}^p}(x_i,x_j).$$

In the above equation, *x*_*i*_, $x_j \in {\Bbb R}^{N_v^p}$ and ${\cal L}^p$ is the normalized Laplacian for ${\cal P}^p{\kern 1pt} \forall p \in P$.

For the problem of finding the optimal mixture of kernels over the different pathway-induced kernels, any supervised MKL algorithm can be used. In this work, a custom version of EasyMKL^41^ was implemented, as it achieves high performance at a low computational cost. EasyMKL is based on the Kernel method for the Optimization of the Margin Distribution (KOMD)^46^ and focuses on optimizing a linear combination of kernels:

24 $$K = \mathop {\sum}\limits_{p = 1}^P {\kern 1pt} w_pK^p,\,w_p \ge 0.$$

In PIMKL, the weights obtained are divided by their sum, as we are interested in evaluating the relative contribution of each kernel. This normalization does not affect the quality of the kernel mixture, which is invariant under positive scalar multiplication. In addition, to account for differences in sub-graph sizes, we force the kernel matrices to have an equal trace, ensuring comparable Gram matrices between different pathways.

It is important to note that PIMKL formulation enables a seamless integration of multi-omics data. Kernels from different data types can be easily generated and added to the mixture. The same applies to multi-modal data integration: kernels generated from other data modalities associated with a specific sample, e.g., histopathology images or clinical records, can be added to the mixture and weighted accordingly to their contribution in the classification problem.

### Reporting summary

Further information on experimental design is available in the Nature Research Reporting Summary linked to this article.

## Supplementary information

Supplementary material of ’PIMKL: Pathway Induced Multiple Kernel Learning’.

Reporting Summary

## Data Availability

All data used in this manuscript is publicly available. Details about cohorts and accession numbers can be found in Tables S1 and S2. Processed data and materials used to produce the results can be downloaded from the following link https://ibm.biz/pimkl-data. PIMKL as a service is freely available on IBM Cloud at the following link https://ibm.biz/pimkl-aas.

## Supplementary information

**Supplementary information** accompanies the paper on the *npj Systems Biology and Applications* website (10.1038/s41540-019-0086-3).
