# Supplementary material for: PIMKL: Pathway-Induced Multiple Kernel Learning
Source: NPJ Syst Biol Appl. 2019 Mar 5;5:8. doi: 10.1038/s41540-019-0086-3 (PMC6401099; doi:10.1038/s41540-019-0086-3)
Supplement: Supplementary file 1 — Supplementary material of ’PIMKL: Pathway Induced Multiple Kernel Learning’. [file 41540_2019_86_MOESM1_ESM.pdf]

Supplementary material of 'PIMKL: Pathway Induced Multiple Kernel Learning'

Matteo Manica, Joris Cadow, Roland Mathis, María Rodríguez Martínez

Supplementary figures and tables

Table S1: **Breast cancer benchmark cohorts.** Brief description of sample counts in the different classes for the cohorts considered in [1] (all Affymetrix Human Genome U133A Array). In GSE4922 and GSE11121 metastasis free survival (dmfs) is considered, in other cohorts relapse free survival (rfs).

**GEOid** [2]	**Patients**	**dmfs/rfs $\leq$ 5 years**	**dmfs/rfs $>$ 5 years**
GSE2034 [3]	286	93	183
GSE1456 [4]	159	34	119
GSE2990 [5]	187	42	116
GSE4922 [6]	249	69	159
GSE7390 [7]	198	56	135
GSE11121 [8]	200	28	154

Table S2: **Breast cancer METABRIC cohort.** Brief description of sample counts in the different classes for the considered data types in the METABRIC (Molecular Taxonomy of Breast Cancer International Consortium) cohort [9].

**Data types**	**Patients**	**Recurred/Progressed**	**DiseaseFree**
Illumina Human v3 microarray (mRNA)<br>Affymetrix SNP 6.0 copy number (CNA)	1890	647	1333

Algorithm S2 PIMKL Cross-validation on METABRIC. Cross-Validation on METABRIC single omics or multi-omics. Given as input: X molecular measurements comprised of a selection of data types T (CNA, mRNA or both) with related clinical labels y, a set of P pathways with a respective pathway for each data type in T and $\lambda = 0.2$ for EasyMKL.

for 100 folds with 20 samples per class in (X_{train}, y_{train}) **do**
    for *type* in T **do**
        learn feature-wise normalization on $X_{type,train}$ and apply to $X_{type,validation}$
    train PIMKL(λ) on $\{k_{\mathcal{L}^p}(X_{train}, X_{train}) : p \in P\}$ and y_{train}
    report kernel weights $\boldsymbol{w}$
    report area under the curve for prediction on $\sum_{p=1}^{P} w_p k_{\mathcal{L}^p}(X_{train}, X_{validation})$

Algorithm S1 PIMKL Cross-validation on [1]. Cross-validation analysis of PIMKL for each of the breast cancer cohorts as suggested in [1] (with internal optimization of parameters). Given as input: X gene expression measurements with related clinical labels y, a set of P pathways and a set of hyper-parameters $\Lambda = \{0,\ 0.1,\ 0.3,\ 0.5,\ 0.7,\ 0.9,\ 1.0\}$ for EasyMKL.

---

for $i \leftarrow 1, 10$ **do**
  for $(X_{train}, y_{train}), (X_{validation}, y_{validation}) \leftarrow$ stratified 10-fold split X, y **do**
    learn feature-wise normalization on X_{train} and apply to $X_{validation}$
    for $(X^{\lambda}_{train}, y^{\lambda}_{train}), (X^{\lambda}_{test}, y^{\lambda}_{test}) \leftarrow$ stratified 3-fold split of X_{train}, y_{train} **do** ▷ parameter grid search with 3-fold cross-validation
      for $\lambda \in \Lambda$ **do**
        train PIMKL(λ) on $\{k_{\mathcal{L}^p}(X^{\lambda}_{train}, X^{\lambda}_{train}) : p \in P\}$ and y^{λ}_{train}
        record prediction accuracy on $\sum_{p=1}^{P} k_{\mathcal{L}^p}(X^{\lambda}_{train}, X^{\lambda}_{test})$
    $\lambda^* \leftarrow$ argmax(mean prediction accuracy over cross-validation)
    PIMKL(λ^*) on $\{k_{\mathcal{L}^p}(X_{train}, X_{train}) : p \in P\}$ and y_{train}
    report kernel weights $\boldsymbol{w}$
    report area under the curve for prediction on $\sum_{p=1}^{P} w_p k_{\mathcal{L}^p}(X_{train}, X_{validation})$
  report mean area under the curve over 10-fold splits ▷ for figure S1 and 3a

---

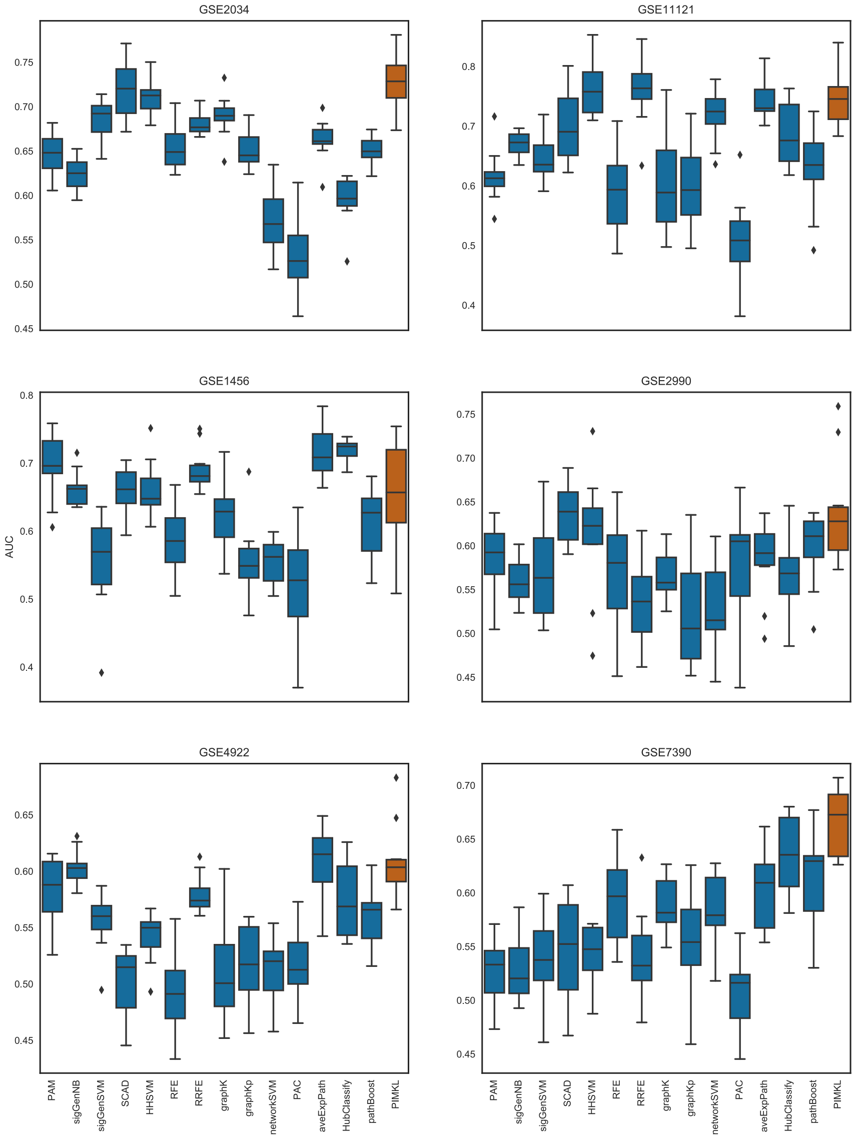

Figure S1: **PIMKL cross-validation AUC.** Box plots of the AUC values for the methods analyzed in [1] (blue) and PIMKL (red). PIMKL clearly outperforms other methods in four out of six data sets. For GSE1456 the performance of PIMKL was close to the other methods average while for GSE11121 PIMKL performance was in the top group. Results are presented as in [1], where each box is drawn from ten (repeats of) mean AUC values over 10-fold cross-validation splits, see algorithm S1.

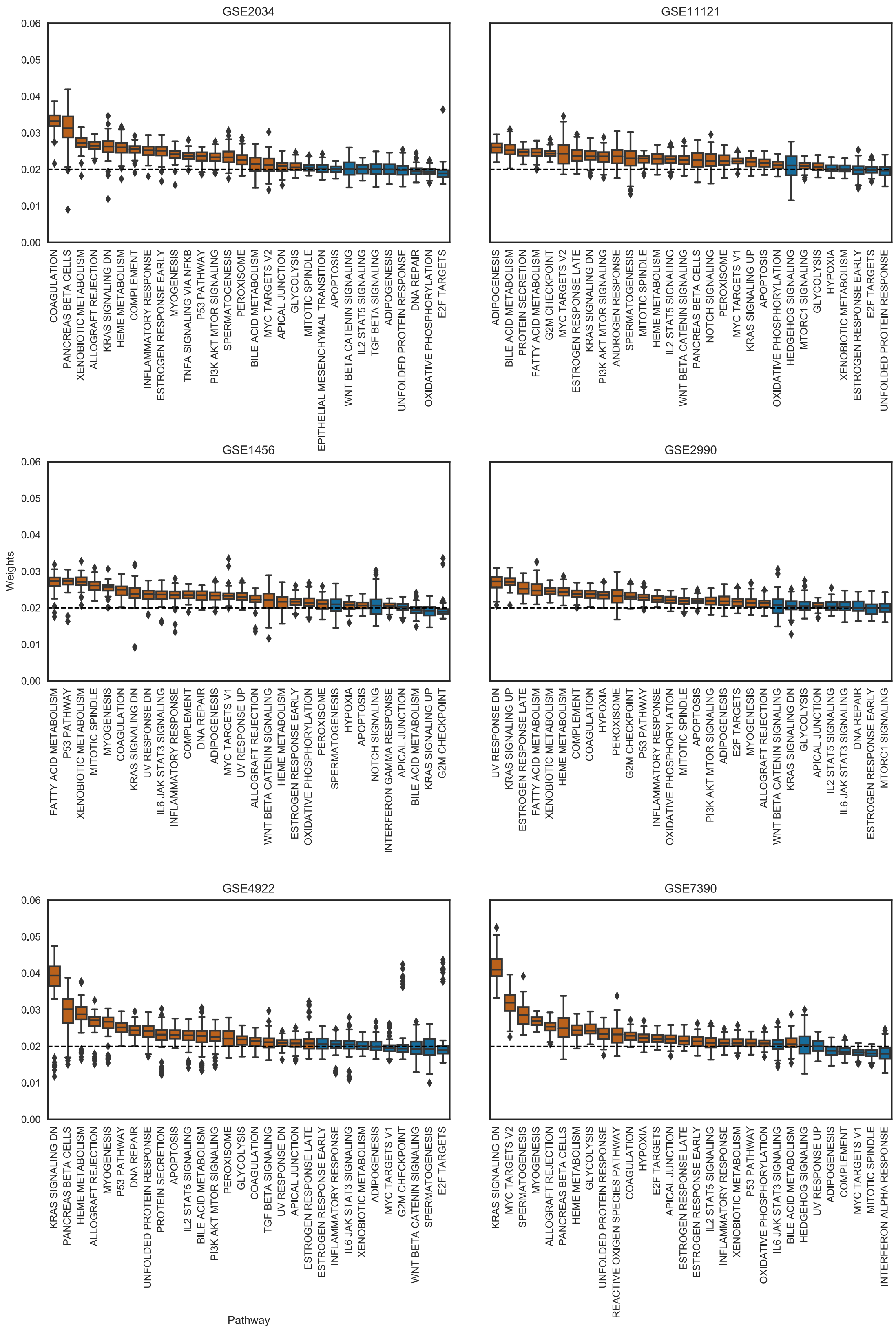

Figure S2: **PIMKL top-30 cross-validation weights in the six cohorts.** The box plots show the weights assigned by PIMKL to the top-30 pathways over cross-validation runs. We report in red the pathways that significantly show high weights over the cross-validation runs, where significance is assessed by performing a one-sample Wilcoxon signed-rank test to compare PIMKL-assigned pathways weights to those resulting from giving each pathway the same weight (see Section 3.1 for details).

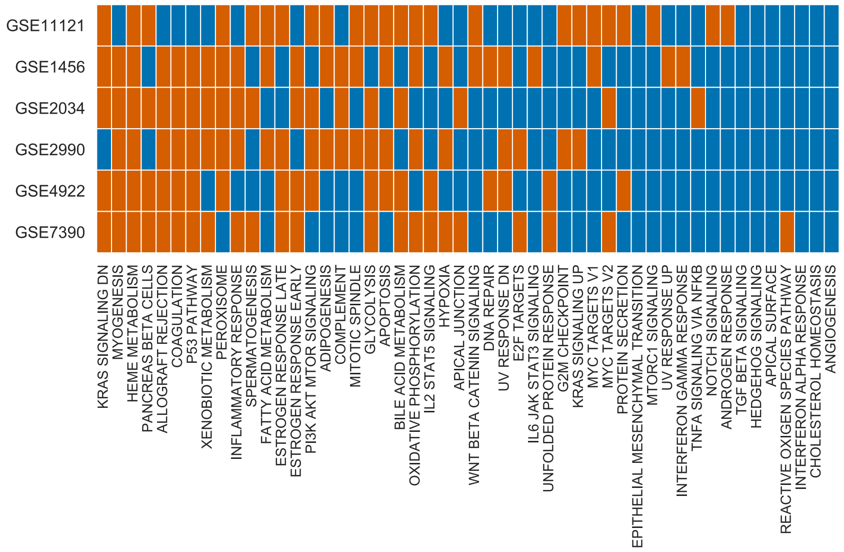

Figure S3: **PIMKL cross-validation weights in the six cohorts.** Significance of weights over 100 cross-validation folds for the 50 hallmark pathways are reported. Significant pathways are colored in red, while non-significant in blue.

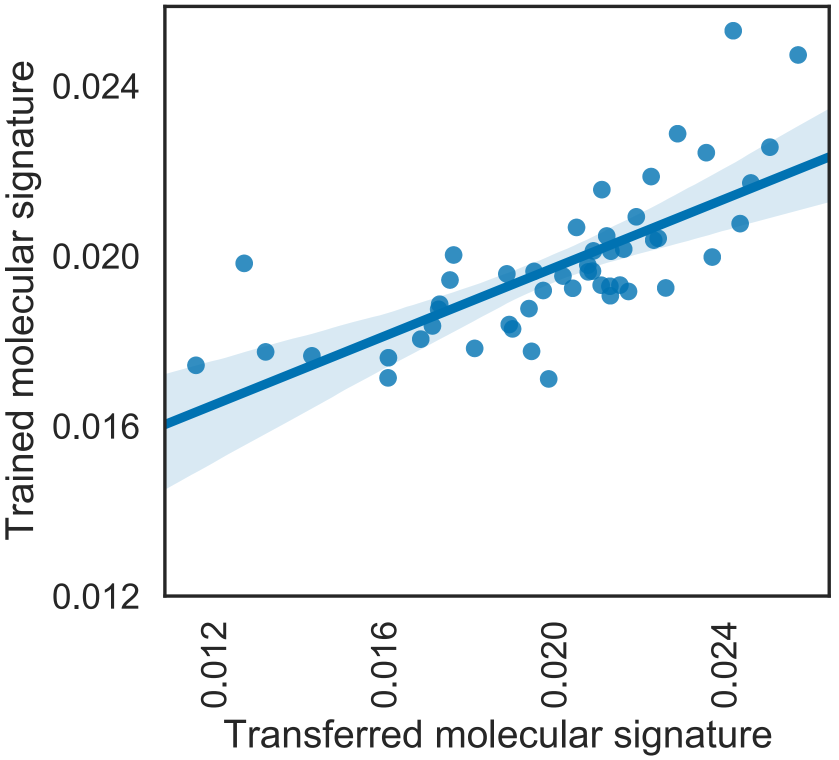

Figure S4: **Regression between trained and transferred signature.** Regression of the pathway weights of the signature obtained from directly training on METABRIC (median over 100 cross-validation folds) against the transferred signature obtained from training on six independent cohorts (each median over 100 cross-validation folds) indicating high correlation of the two signatures.

References

1. Cun, Y. & Fröhlich, H. Prognostic gene signatures for patient stratification in breast cancer-accuracy, stability and interpretability of gene selection approaches using prior knowledge. *BMC bioinformatics.* http://www.biomedcentral.com/content/pdf/1471-2105-13-69.pdf (2012).
2. Barrett, T. *et al.* NCBI GEO: Archive for functional genomics data sets - Update. *Nucleic Acids Research* **41.** ISSN: 03051048. doi:10.1093/nar/gks1193 (2013).
3. Wang, Y. *et al.* Gene-expression profiles to predict distant metastasis of lymph-node-negative primary breast cancer. *Lancet* **365,** 671–679. ISSN: 01406736 (2005).
4. Pawitan, Y. *et al.* Gene expression profiling spares early breast cancer patients from adjuvant therapy: derived and validated in two population-based cohorts. *Breast Cancer Research* **7,** R953. ISSN: 1465-542X (2005).
5. Sotiriou, C. *et al.* Gene expression profiling in breast cancer: Understanding the molecular basis of histologic grade to improve prognosis. *Journal of the National Cancer Institute* **98,** 262–272. ISSN: 00278874 (2006).
6. Ivshina, A. V. *et al.* Genetic Reclassification of Histologic Grade Delineates New Clinical Subtypes of Breast Cancer. *Cancer Research* **66,** 10292–10301. ISSN: 0008-5472 (2006).
7. Desmedt, C. *et al.* Strong time dependence of the 76-gene prognostic signature for node-negative breast cancer patients in the TRANSBIG multicenter independent validation series. *Clinical cancer research : an official journal of the American Association for Cancer Research* **13,** 3207–14. ISSN: 1078-0432 (June 2007).
8. Schmidt, M. *et al.* The humoral immune system has a key prognostic impact in node-negative breast cancer. *Cancer Research* **68,** 5405–5413. ISSN: 00085472 (2008).
9. Curtis, C. *et al.* The genomic and transcriptomic architecture of 2,000 breast tumours reveals novel subgroups. *Nature* **486,** 346 EP - (Apr. 2012).
